# Geometric properties of musical scales constitute a representational primitive in melodic processing

**DOI:** 10.1016/j.isci.2025.113701

**Published:** 2025-10-09

**Authors:** Omri Raccah, Michael Seltenreich, Claire Pelofi, Fred Lerdahl, David Poeppel

**Affiliations:** 1 Department of Psychology, Yale University, New Haven, CT, USA; 2 Department of Psychology, New York University, New York, NY, USA; 3 Anthropic, San Francisco, CA, USA; 4 Department of Music, New York University, New York, NY, USA; 5 Center for Language, Music, and Emotion (CLaME), New York University, New York, NY, USA; 6 Department of Music, Columbia University, New York, NY, USA; 7 Max Planck Society, Munich, Germany

**Keywords:** Music, Cognitive Psychology, Cognitive Science

## Abstract

Uncovering the mental representations underlying symbolic domains is a long-standing goal of cognitive science. Music, a relatively unexplored aspect of cognition, presents a unique testbed for understanding the format of symbolic representations. Theoretical accounts have emphasized the geometric structure underlying musical scales, yet empirical work directly engaging with these structures remains limited. Based on data collected from 961 participants across four experiments, we demonstrate that certain geometric structures strongly modulate perceptual sensitivity to out-of-scale notes in melodies. Furthermore, we show that geometric regularity predicts sensitivity to note deviations across diverse musical structures. Crucially, these effects persist even in an entirely unfamiliar tuning system, suggesting the role of specific geometric properties independent of learned cultural associations. Our findings situate scale geometry as a critical, time-invariant representation in melodic processing and reveal organizational principles that may underlie mental representations across domains.

## Highlights

• Data from hundreds of participants elucidates how musical scales shape perception
• The geometry underlying scales influences perceptual sensitivity to note deviations
• Geometric regularity predicts perceptual sensitivity across diverse structures
• Effects persist in the absence of a familiar tuning system

## Graphical abstract

**Figure:**
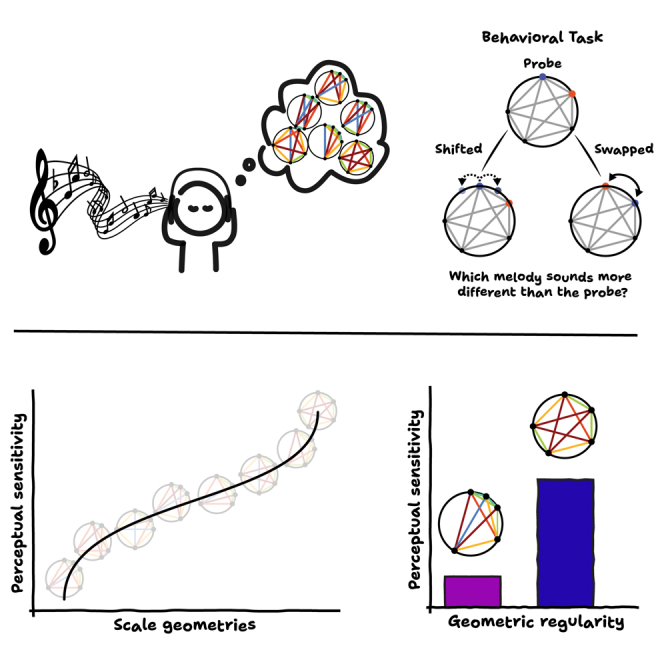

## Introduction

A long-standing goal of cognitive science is to uncover the computations and mental representations across symbolic domains, including language, music, and mathematics.^1^^,^^2^^,^^3^^,^^4^^,^^5^ The integration of theoretical frameworks with empirical perspectives has led to significant strides in understanding how language is represented in the human brain^6^^,^^7^. Like language, music contains a complex, multilayered constituent structure. In language, syllables combine into words, and words combine into phrases. In music, individual notes combine into motifs, and motifs combine into musical phrases. Critically, while the constituents and terminal elements themselves are domain-specific, properties such as hierarchical relations and the notion of constituency itself can be considered domain-general.^8^^,^^9^^,^^10^ Despite its similarly rich theoretical foundation, the cognitive basis of many formally specified concepts in music has yet to be empirically investigated. As such, music presents tremendous potential to provide insights into the informational content and format of symbolic representations.

One promising entry point for exploring the cognitive foundations of musical structure lies in the study of musical scales. While musical traditions exhibit extraordinary diversity, certain note combinations have persisted throughout history and transcended cultural boundaries.^11^^,^^12^^,^^13^^,^^14^ The cross-cultural reemergence of certain note combinations – for instance, that of the pentatonic scale – suggests a potential cognitive basis for these structures. Theoretical frameworks have employed geometric approaches to understand the structural properties of musical scales,^15^^,^^16^^,^^17^^,^^18^^,^ facilitating direct comparison with other symbolic domains. Notably, certain geometric properties, e.g., symmetrical structures, enhance perceptual abilities across sensory modalities and diverse cognitive tasks.^10^^,^^19^^,^^20^^,^^21^^,^^22^ Likewise, corpus analyses have shown that scales with certain geometric features underlie the majority of musical systems globally.^12^^,^^13^^,^^23^ Furthermore, the relational (intervallic) structure of scales aligns with established models of schematic representations in long-term memory (for review, see^24^). Therefore, musical scales offer a foundation for investigating organizational principles of mental representation and how specific structural features influence the processing of complex sequential information.

The musical scale serves as the basis for most pitch-based research in music cognition. For instance, studies on the hierarchical organization of notes explore their functional roles within a scale (i.e., research on tonality^25^^,^^26^^,^^27^^,^^28^^,^^29^^,^^30^^,^^31^^,^^32^^,^^33^^,^^34^). Similarly, research into the time-varying and statistical components of melodic processing examines these phenomena in relation to notes belonging to a scale.^35^^,^^36^^,^^37^^,^^38^^,^^39^ These paradigms have also provided key insights into the shared neural mechanisms of language and music.^40^^,^^41^^,^^42^ While such studies rely on scales, their primary focus is on higher-level musical elements rather than the direct influence of scales themselves. Furthermore, most studies use only a limited set of culturally familiar scales. A comprehensive and direct investigation of scales could bridge findings across representational constituents in melodic processing. To address this challenge, our work seeks to understand how structural properties of scales may facilitate perceptual sensitivity to out-of-scale note violations in melodies.

**Figure 1:**
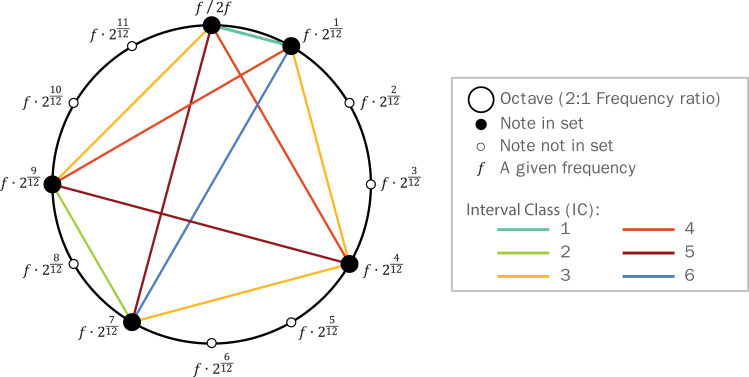
Illustrating the geometric structure of musical scales The underlying structure of musical scales is defined by the intervallic structure established by a set of notes. These structures are constructed by using a subset of notes from the 12 available notes in standard tuning, commonly represented in a circular pattern representing the span of an octave (or other circular arrangements such as a tritave). Note that the illustrated set serves as an exemplar, selected for its inclusion of multiple interval classes. This set has 1 instance of IC1 (“interval class 1”) in teal, 1 of IC2 in green, 3 of IC3 in yellow, 2 of IC4 in orange, 2 of IC5 in red, and 1 of IC6 in blue. Importantly, different combinations of notes can form the same set, as long as the relational structure is maintained (relative distances or intervals across notes).

Throughout this work, we analyze the geometric structure of scales using the foundational framework of musical set theory.^15^^,^^16^^,^^17^^,^^18^^,^^43^ Within this framework, musical notes are represented using numerical values, allowing us to engage with these musical structures from the vantage point of geometry. As shown in Figure 1, a set can be depicted as a collection of graph nodes (representing notes) distributed along the circumference of a circle (representing an octave; frequency ratio 2:1). The nodes are connected by edges representing musical intervals (the frequency ratios between two notes). Importantly, what defines a given set is its relational structure as opposed to its specific note identities.^44^ As such, different notes can give rise to the same set as long as their overall intervallic structure is maintained.

We conducted four behavioral experiments to investigate the influence of scale structures on melodic processing. We hypothesized that certain scale structures would determine the degree to which a given structure can facilitate sensitivity to note deviations in melodies. In Experiment 1, we examine the effect elicited across two emblematic Western musical structures and investigate how these effects are modulated by melody length. Experiment 2 examines a larger universe of musical sets (all 66 five-note sets available in standard tuning) and tests how certain geometric features modulate perceptual sensitivity. In Experiment 3, we test the impact of perfectly even sets, characterized by an evenly distributed arrangement of notes across the octave. This experiment elucidates the effects associated with this distinct class of sets, which minimize the potential impact of hierarchical or tonal organization.^45^^,^^46^^,^^47^ Finally, in Experiment 4, we provide evidence that the geometric structure underlying scales impacts perceptual sensitivity even in an unfamiliar tuning system (tritave instead of octave-based). Together, these experiments provide evidence for the geometry of scales as a critical instance of mental representation in melodic processing.

## Results

**Figure 2:**
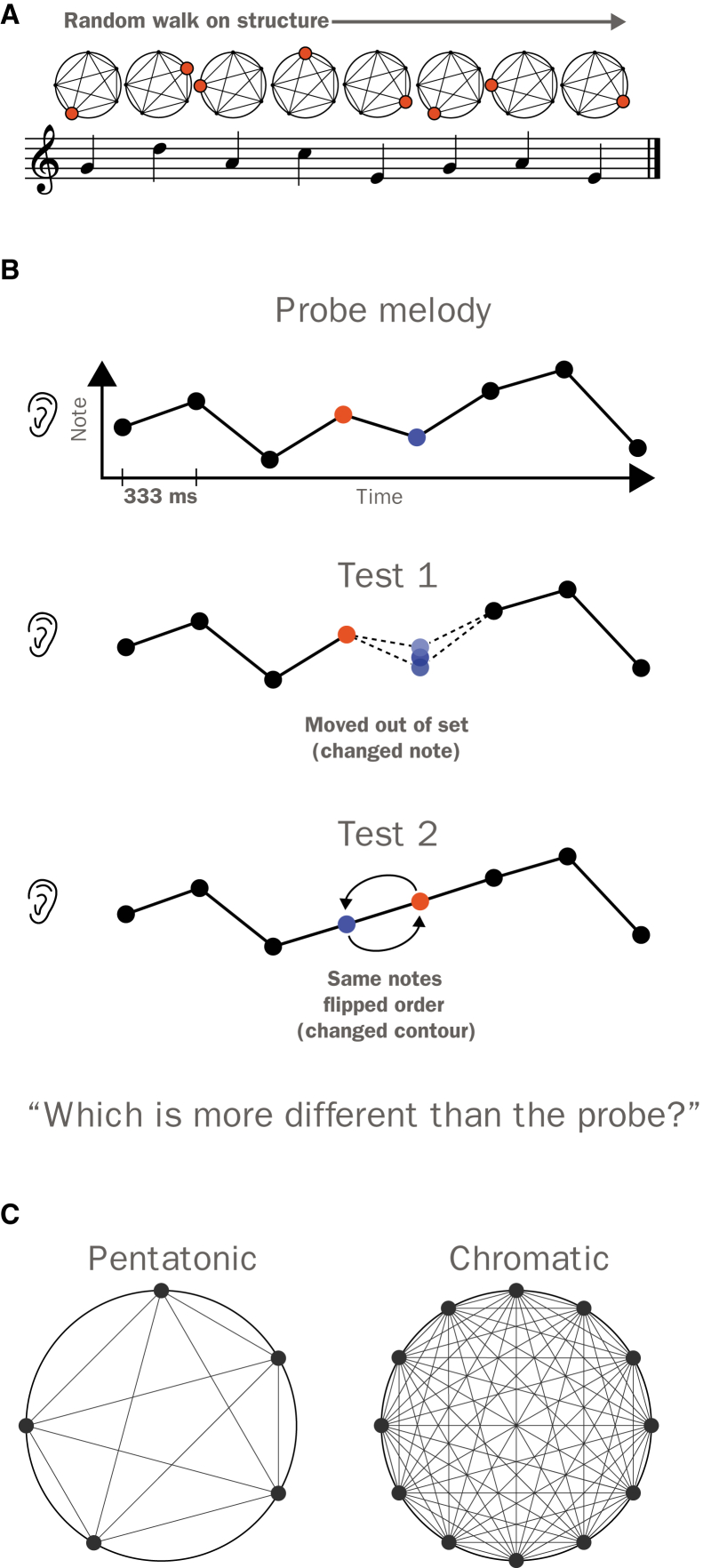
Experimental procedures and task paradigm (A) Melodies used in this study were generated using a pseudo-random walk procedure within a specific musical set. (B) During each block, participants listened to a probe melody followed by two test melodies. The test melodies were identical to the probe melody, with the exception that one introduced a note that never appeared in the probe (*note-deviation condition*), while the other served as a control by flipping the order of the middle notes while preserving the original pitches (contour-deviation condition). Immediately after hearing the three melodies, participants were asked to judge which melody sounded more different from the probe melody. It is important to note that this task schematic demonstrates melodies of length 8. However, two additional cohorts were administered to this paradigm with melody lengths of 12 and 16, respectively. (C) In Experiment 1, participants heard melodies generated from the pentatonic set (pitches 0, 2, 4, 7, and 9) or the chromatic set, comprising two experimental conditions, as depicted in this panel.

Across experiments, melodies were generated using a pseudo-random procedure, controlling for pitch range, consecutive repetitions, melodic leaps, and other relevant factors (EDO.js toolbox,^48^; see STAR Methods for a detailed description of stimulus generation). This random-walk procedure allowed us to examine the influence of distinct relational structures on perception, eliminating the effect of known temporal associations (Figure 2A). In each trial, a set was realized at a random transposition (i.e., the base frequency of the set is chosen at random; see Figure 1) to avoid habituation to certain pitch frequencies. In this way, what defines a set is not dependent on the identity of its notes, but on its relational structure. Critically, we generated melodies constrained to a fixed range (a single octave or tritave in Experiment 4; see STAR Methods) to avoid the potential influence of octave displacement.^49^^,^^50^^,^^51^^,^^52^

In all experiments, participants took part in a behavioral protocol to capture the influence of a given set’s geometry on sensitivity to note deviations in melodies (Figure 2B). This task extends previous paradigms that compare contour and pitch representations in melodic processing.^53^^,^^54^^,^^55^ In each trial, participants listened to a probe melody generated from a particular set, followed by two test melodies. The test melodies were identical to the probe melody, with the exception that one introduced a note that never appeared in the probe (*note-deviation condition*), while the other served as a control by flipping the order of the middle notes while maintaining the original note collection (*contour-deviation condition*). As such, note deviations were intended to “break out” of the note collection, providing a metric for the perceptual effect associated with the set as an overarching context. On the other hand, contour deviations did not change the identity of the members making up a melody but instead only changed the overall shape of the melody. On each trial belonging to a particular set, participants were asked to indicate which of the two test melodies sounded more different than the probe melody. The extent to which participants indicated that contour deviations were more different reflects sensitivity to overall shape changes in the melodies. In contrast, the extent to which participants indicated that note deviations were more different reflects sensitivity toward a unified view of the overall set structure. Participants also had the option to report hearing no difference between the test melodies; these responses were excluded from the subsequent analyses (See STAR Methods section).

### Experiment 1: A prevalent set across different melody lengths

In Experiment 1, we tested the impact of a prevalent set on the processing of melodies. We compared behavioral responses for the pentatonic set relative to the chromatic set (Figure 2C). The pentatonic set was chosen because of its widespread use cross-culturally (Western and non-Western traditions^56^). Therefore, the pentatonic set provides an exemplary structure to investigate the degree to which a well-established set modulates perception. At the other extreme, the chromatic set, the collection of all available notes in the modern Western musical system (12 equal divisions of the octave; 12-EDO), was chosen, given that it is a superset of all possible sets. As such, the chromatic set represents an appropriate control in that it can be considered a “no-set condition.” In doing so, we tested this hypothesis across a variety of melody lengths (8-note, 12-note, and 16-note) in three different cohorts to comprehensively characterize the behavioral effects.

**Figure 3:**
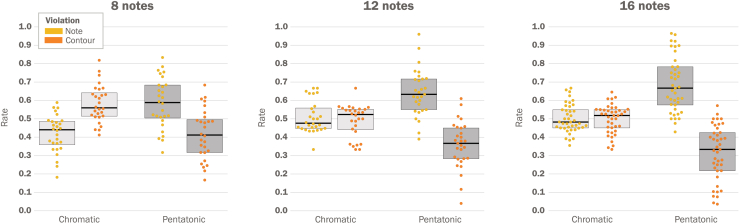
Experiment 1 results: response bias for a prevalent set across melody lengths We compared response bias for the pentatonic set and the chromatic set for three melody lengths, probed across three separate cohorts: 8-note (*N* = 28), 12-note (*N* = 29), and 16-note (*N* = 41). In 8-note melodies, the pentatonic set elicited a significant preference bias for note violations relative to contour violations, while the chromatic set showed a significant opposite pattern (i.e., a preference for contour violations). Critically, 12-note and 16-note melodies showed an even stronger preference for note violations than for shorter melodies (8-note melodies). Furthermore, the response patterns elicited by the chromatic set for contour violations were no longer significant for cohorts who were exposed to longer melodies. The data suggest that evidence accumulated across individual notes toward a time-invariant set structure (i.e., the prevalent pentatonic set) serves to drive a sense of membership during melodic processing. Dots, individual participants; black lines, mean across participants; gray shaded regions, standard deviation.

Three cohorts of participants (*N* = 98 total, after exclusion) were assigned to perform the task with different melody lengths: 8-note (*N* = 28), 12-note (*N* = 29), and 16-note (*N* = 41). The behavioral results for the three cohorts are shown in Figure 3. Although the figure illustrates the rate of each violation type, we assessed statistical significance using the signed difference between the rate of note- and contour-violations, as a measure of bias toward note deviations. We used a two-way mixed analysis of variance (mANOVA) to analyze the between-subjects melody length effects (8-note, 12-note, and 16-note) and the within-subjects condition type effects (pentatonic and chromatic). We found a significant main effect for condition type (*F*(1, 94) = 125.07, *p* < 0.001, $\eta_{p}^{2}$ = 0.57) as well as a significant main effect for melody length (*F*(2, 94) = 8.73, *p* < 0.001, $\eta_{p}^{2}$ = 0.16). These findings reflect significantly enhanced sensitivity to note deviations in the pentatonic set relative to the chromatic set. Furthermore, perceptual sensitivity to note deviations increased across cohorts for longer melody lengths. We did not find a significant melody length by condition type interaction (*F*(2, 94) = 0.92, *p* = 0.4, $\eta_{p}^{2}$ = 0.02). For 8-note melodies, follow-up one-sample *t-*tests revealed opposing results across sets, such that the pentatonic structure elicited significant sensitivity to note deviations (*t*(27) = 3.28, *p* = 0.003, Cohen’s *d* = 0.62), while the chromatic set elicited significant response bias toward contour deviations (*t*(27) = −4.23, *p* < 0.001, Cohen’s *d* = −0.8). For 12- and 16-note melodies, we similarly found a significant response bias elicited by the pentatonic set for note deviations (12 note: *t*(28) = 6.04, *p* < 0.001, Cohen’s *d* = 1.12; 16 note: *t*(40) = 7.92, *p* < 0.001, Cohen’s *d* = 1.24). The effect of contour deviation was not significant for the chromatic structure for these longer melody lengths (12 note: *t*(28) = 0.54, *p* = 0.59, Cohen’s *d* = 0.1; 16 notes: *t*(40) = 0.08, *p* = 0.94, Cohen’s *d* = 0.01).

Next, we used this dataset to test whether these effects were present in non-musicians. We aggregated the data for 12-note and 16-note melodies for participants with self-reported no musical training and no experience playing a musical instrument (*N* = 18). We focused on 12- and 16-note melodies due to their comparable behavioral results. Our analysis revealed the same pattern of results persisted, with a significant preference for note-deviations in the pentatonic set (*t*(17) = 3.17, *p* = 0.006, Cohen’s *d* = 0.75; Figure S1A), and no significant preference for the chromatic set (*t*(17) = −1.34, *p* = 0.2, Cohen’s *d* = −0.32; Figure S1A). These findings demonstrate that these effects can be reliably observed in non-musicians.

### Experiment 2: The entire universe of 5-note sets in standard tuning

Exp. 1 revealed that the widely prevalent pentatonic set facilitates a strong sense of membership within note collections. This finding raises the question of which specific structural properties underlie this perceptual phenomenon. For instance, it is necessary to investigate whether the findings from Exp. 1 are due solely to differences in the number of notes in the pentatonic and chromatic sets (i.e., 5 notes in the pentatonic set and 12 notes in the chromatic set). This would indicate a potential difference in processing demands based on set cardinality (i.e., the number of notes within a set). Alternatively, the observed effects may be linked to the precise structural characteristics inherent to these sets (e.g., their respective intervallic structure).

**Figure 4:**
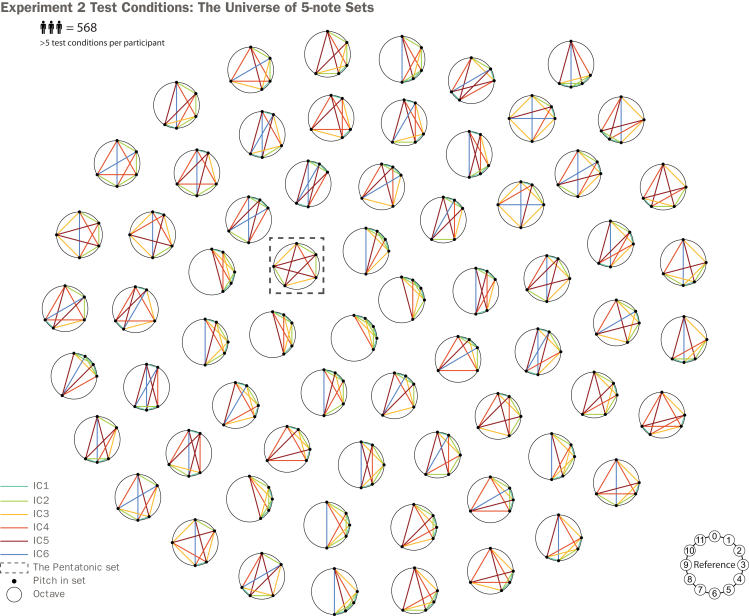
The universe of all five-note musical sets used in Experiment 2 In Experiment 2, the same task paradigm introduced in Exp. 1 is executed (Figure 2B). We generated melodies across the entire universe of five note sets (in standard tuning, i.e., 12-EDO). Among these five-note sets is the pentatonic set, which was used in Exp. 1 for its prevalence in music cross-culturally. Note that the sets are plotted in numerical order of their constituent notes and are not intended to give an intuition for our hypotheses.

Exp. 2 was conducted to determine which structural properties may have contributed to the perceptual phenomenon observed in Exp. 1. This experiment employed the same behavioral paradigm but with a larger cohort (*N* = 568, post-exclusion). Here, we tested all 66 five-note sets in standard tuning (including the pentatonic set). This space captures a wide range of geometric configurations, as illustrated in Figure 4. The choice of using 5-note sets was motivated by the cardinality commonly used in musical sets cross-culturally.^11^ Each participant performed the task, interacting with six distinct and randomly chosen sets, with the option to complete the task multiple times. Notably, 12-note melodies were used to generate the stimuli for this task, given the strong response bias for note deviations with this melody length in Exp. 1 (see STAR Methods section). This comprehensive dataset allowed us to investigate which structural properties contribute to the effects reported in Exp. 1.

**Figure 5:**
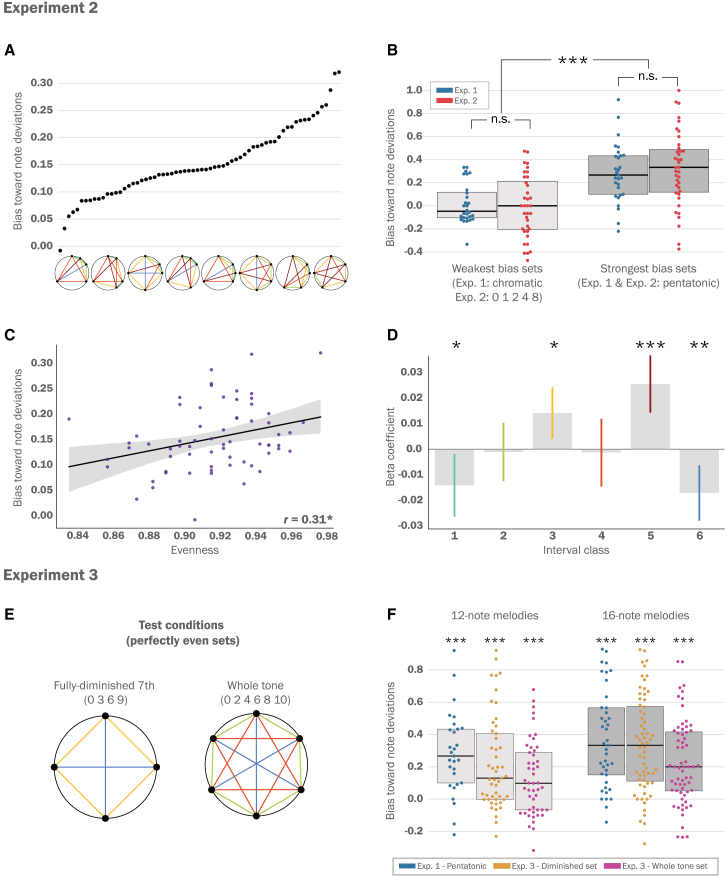
Experiments 2 and 3 results: structural features driving perceptual sensitivity (A) Distribution of scores representing participants’ bias toward note deviations across the 66 sets in Exp. 2. The x-axis displays set structures evenly sampled from this distribution. The scores were modulated along a wide continuum, with certain sets having little to no overall effect, while others elicited a bias that was twice the magnitude of contour violations. (B) The response pattern for the chromatic set in Exp. 1 was not significantly different from that of the lowest-performing set in Exp 2. Furthermore, the pentatonic set, which was the highest-performing set in Exp. 2, performed similarly across the two experiments (two-sample t-test). (C) The evenness of a set was positively correlated with participants’ average bias across sets (Pearson correlation). (D) Using a multiple regression analysis, we find that the interval vector of a set, or the count of each interval class, robustly predicts the scores across sets. Through examining the coefficients for each interval, we found that IC1 and IC6 display a significant negative relationship with the scores, while IC3 and IC5 display a positive relationship (permutation test, 10,000 permutations). Error bars represent 95% confidence intervals. (E) The melodies in Exp. 3 were generated from two perfectly even sets consisting of 4 notes and 6 notes, respectively. (F) For both melody lengths, the perfectly even sets tested in Exp. 3 elicited a significant bias toward note deviations, with the diminished 7^th^ set showing a comparable magnitude to the pentatonic set in Exp. 1 and Exp. 2. Panels B and F: Dots, individual participants; black lines, mean across participants; gray shaded regions, standard deviation. ∗*p* < 0.05. ∗∗*p* < 0.01. ∗∗∗*p* < 0.001.

To examine sensitivity to note deviations, we computed the average response bias for these melodies across participants for each of the 66 five-note sets (i.e., the signed difference between the response rate for note violations and contour violations). These scores served as a measure of perceptual sensitivity to note deviations afforded by each set. This analysis revealed a wide distribution, with certain sets eliciting a robust sensitivity for note deviations, while others exhibited little to no bias (Figure 5A). Table S1 provides a detailed description of set properties and their respective average scores. The findings show that the pentatonic set elicited the highest average response bias for note deviations compared to all other 5-note sets. Importantly, the extent of note sensitivity elicited by the pentatonic set did not significantly differ from the results of Exp. 1, thus replicating these findings in a separate cohort (*t*(66) = −0.5, *p* = 0.62, Cohen’s *d* = −0.12; Figure 5B). Interestingly, the set associated with the weakest sensitivity to note deviations (referred to as having the “weakest bias”) was not significantly different from the chromatic set observed in Exp. 1 (*t*(63) = 0.45, *p* = 0.66, Cohen’s *d* = 0.11; Figure 5B). Similar to Exp. 1, aggregating participants for the three highest and lowest-performing sets, we found that non-musicians exhibited a significant bias toward note deviations in the high-performing sets (*t*(17) = 2.42, *p* = 0.028, Cohen’s d = 0.59), while preference for note deviations in the low-performing sets was not statistically significant (*t*(23) = 0.15, *p* = 0.88, Cohen’s *d* = 0.03; Figure S1B).

We also collected familiarity ratings for melodies generated by all 66 sets in this experiment. These ratings were collected from a separate cohort of participants (*N* = 147, after exclusions; see STAR Methods). While we found no significant correlation between the average median familiarity ratings and note sensitivity across sets (*R* = 0.23, *p* = 0.06), other subjective measures accounted for significant variability (Figure S3). Importantly, we address the influence of familiarity with Western musical systems more directly in Experiment 4.

### Geometric and structural features driving perceptual sensitivity

The results from Exp. 2 show that scores span a wide continuum. This continuum indicates the varying degree to which sets can impart a sense of note membership. Furthermore, specific 5-note sets showed no significant bias toward note deviations compared to the chromatic set, indicating that the bias observed with the pentatonic set in Exp. 1 cannot be attributed solely to the number of notes across sets. These findings raise the question of which structural feature(s) drive sensitivity for note deviations across 5-note sets. Although one could consider a range of hypotheses regarding such features, we test two structural features that are particularly well motivated from theoretical and cognitive standpoints.

First, we tested the hypothesis that this continuum can be captured by how evenly pitches are distributed along the octave, a property referred to as *evenness* in musical set theory.^18^^,^^57^ It has been suggested that sets with a greater degree of evenness lead to less perceptual ambiguity for set members by maximizing the overall distance between individual notes.^15^^,^^46^ To our knowledge, empirical work has yet to directly test this hypothesis. We quantified the evenness of each of the 66 sets by comparing its note positions to a theoretical perfectly even 5-note set (see STAR Methods section). In line with theoretical proposals, we found a significant correlation between evenness and sensitivity to note deviations across all sets (R = 0.31, *p* = 0.012; Figure 4C).

We next tested the impact of each set’s interval classes, the theoretical structural constituents of a set. These intervals represent unique and recognizable musical primitives that are fundamental to the way humans perceive music.^58^^,^^59^ It is because we regard the intervallic relationships as constitutive rather than the notes themselves that allows us to identify a song or a melody across different keys (often without even knowing that a change of key took place;^60^). Notably, each such interval has multiple levels of interpretation. While this work interacts with intervals musically as structural primitives, each interval also carries a psycho-acoustic interpretation that modulates low-level auditory processing.^61^ The six interval classes are marked as IC1 to indicate an interval class spanning 1 semitone, through IC6 referring to an interval class spanning 6 semitones, accordingly.

To understand the impact of intervals on participants’ behavior, we performed a multiple ridge regression analysis. The intervals for each set were tallied and used as predictors in the regression model. The dependent variable was the average sensitivity toward note deviations for each set across participants. The model achieves a robust prediction of note sensitivity across the 66 sets (R = 0.62, *p* < 0.001; permutation test). We tested whether the coefficients for each interval were significantly above a null distribution to understand the impact of distinct intervallic relationships. We found that IC1 (minor seconds and major sevenths) and IC6 (augmented fourths and diminished fifths) showed a significant negative relationship with the average sensitivity to note deviations (IC1: *B* = −0.014, 95% CI (−0.001, −0.027), *p* = 0.031; IC6: *B* = −0.017, 95% CI (−0.005, −0.029), *p* = 0.009; permutation test), while IC5 (perfect fourths and fifths) and IC3 (minor thirds and major sixths) showed a significant positive relationship (IC5: *B* = 0.025, 95% CI (0.038, 0.013), *p* < 0.001; IC3: *B* = 0.014, 95% CI (0.024, 0.005), *p* = 0.03; permutation test). Other interval classes did not show a significant effect (*p* > 0.05; permutation test).

### Experiment 3: Perfectly even sets

To eliminate the potential influence of tonality, understood as the hierarchical organization of notes,^31^^,^^32^^,^^34^ we conducted a third experiment aimed at investigating two sets that cannot support tonality. Specifically, we used two sets that have an even distribution of notes within an octave: the fully diminished 7^th^ set (4 notes) and the whole-tone set (6 notes; Figure 5E). These sets lack a fundamental geometric property known as uniqueness, which denotes distinct relationships between notes. Uniqueness is a prerequisite for the emergence of hierarchical structure.^45^^,^^46^^,^^47^ We hypothesize that sensitivity toward note deviations will remain significant for these sets, suggesting that tonality alone does not drive these effects. This prediction could not be tested using the 5-note sets from Exp. 2, as they all have distinctive relationships. The sets used in this experiment, which contain no instances of IC5 and are maximally even, also serve as a test of whether these features are essential for eliciting the effects observed in Exp. 2. The experimental procedures followed the same protocols as in Exp. 1. To examine the influence of melody length, we conducted the experiment in two separate cohorts with 12-note melodies (*N* = 49; post-exclusion) and 16-note melodies (*N* = 64; post-exclusion; see STAR Methods section).

For 12-note melodies, we observed a significant bias toward note deviations in both even sets (fully diminished 7^th^ set: *t*(48) = 5.47, *p* < 0.001, Cohen’s *d* = 0.78; whole-tone set: *t*(48) = 3.89, *p* < 0.001, Cohen’s *d* = 0.56; Figure 5F). The diminished set was statistically indistinguishable from the pentatonic set in Exp. 1 (*t*(76) = 0.89, *p* > 0.05, Cohen’s *d* = 0.21). In contrast, the whole-tone set exhibited a significantly weaker bias than the pentatonic set (*t*(76) = 2.79, *p* = 0.007, Cohen’s *d* = 0.65), suggesting a potential effect of cardinality. For 16-note melodies, both sets elicited significant overall biases toward note deviations (fully diminished 7^th^ set: *t*(63) = 9.12, *p* < 0.001, Cohen’s *d* = 1.14; whole-tone set: *t*(63) = 6.98, *p* < 0.001, Cohen’s *d* = 0.87; Figure 5F). Similar to 12-note melodies, the fully diminished 7^th^ set elicited a bias that was not significantly different from the pentatonic set in Exp. 1 (*t*(103) = 0.52, *p* > 0.05, Cohen’s *d* = 0.01), while the whole-tone set showed a significantly weaker bias toward note deviations (*t*(103) = 2.56, *p* = 0.018, Cohen’s *d* = 0.51).

These findings suggest that evenness plays a key role in modulating perceptual sensitivity, irrespective of a potential role of tonality in this process. Furthermore, these findings extend to non-musicians, who showed a significant bias toward note deviations for both the fully diminished 7^th^ set (*t*(31) = 4.3, *p* < 0.001, Cohen’s *d* = 0.77) and the whole-tone set (*t*(31) = 4.26, *p* < 0.001, Cohen’s *d* = 0.77), as shown in Figure S1C.

### Experiment 4: Sets evaluated in an unfamiliar tuning system

When examining the impact of various structural properties across sets, it is important to keep in mind that familiarity may play a role in shaping the results. Indeed, prior exposure is an integral part of consolidating a musical structure in long-term memory and cannot be completely separated when examining the impact of structural features. To address the role of familiarity, we sought to understand whether the relational structure of scales can capture participants’ behavior in an unfamiliar tuning system. Instead of an octave (2:1 frequency ratio), we split a *tritave* (3:1 frequency ratio) into twelve logarithmically equal segments to produce an unfamiliar tuning system (henceforth “UT”). Using the tritave as the basis of the tuning system markedly deviates from traditional Western musical systems, which rely on an octave. For this reason, the tritave has been increasingly used in music cognition research to mitigate the influence of familiarity in Western listeners (e.g.,^62^^,^^63^^,^^64^^,^^65^). We encourage the reader to listen to the melodies (see *Melody Examples* folder in OSF Project: https://osf.io/phj8m/).

In this experiment, we applied the same behavioral protocol with two 5-note sets constructed within the UT system (*N* = 35, post-exclusion; see STAR Methods). Specifically, we instantiated the pentatonic set within the tritave tuning system. This set maintained the same relational structure as in previous experiments while utilizing notes that are entirely unfamiliar to participants. As a control, we used the lowest performing 5-note set from Exp. 2 (with regards to facilitating a sensitivity to note deviations), instantiated within the span of a tritave (notes: 0 1 2 4 8). The stimulus generation procedures and task design followed the same protocols as Exp. 1 using 12-note melodies (see STAR Methods). We hypothesized that the pentatonic set would elicit a greater sensitivity to note deviations compared to the control set in this tuning system.

Following our previous analyses, we compared the average bias toward note deviations across the two structures. As predicted, the pentatonic elicited a significantly greater bias toward note deviations compared to the control (*t*(34) = 2.67, *p* = 0.012, Cohen’s *d* = 0.38; paired t-test; Figure S4). This effect was especially pronounced for participants who report zero years of prior musical training and no experience playing a musical instrument (*t*(10) = 3.41, *p =* 0.008, Cohen’s *d* = 1.07; paired t-test; Figure S4). A larger sample of non-musicians will be needed to fully explain the effect of musicality in this unique tuning system.

## Discussion

We investigated how the geometric structures underlying musical scales impact melodic processing. To do so, we used musical set theory, a central branch of music theory, to engage with these structures quantitatively.^66^ We posit that these schematic structures constitute a particular instance of a mental representation that serves a central function in music perception. Specifically, we predict that scale geometry provides a context during music listening that determines which notes belong, and which do not. To test this hypothesis, we designed a behavioral protocol that implicitly tests participants’ sensitivity toward out-of-set note deviations.

In Exp. 1, we show that a prevalent set exerts a significant modulatory effect on our sensitivity toward note deviations (Figures 2C and 3). Furthermore, we find that this effect interacts with melody length such that sensitivity is enhanced in longer melodies. This may indicate that individual notes provide accumulated evidence toward a unified scale representation. The findings from Exp. 1 provide compelling evidence that a prevalent set strongly modulates perceptual sensitivity across note members. Notably, our findings showcase a phenomenon that is not readily explained using predictive coding frameworks.^38^^,^^39^ Instead, the effects of these time-invariant structures may be best captured by symbolic accounts.^10^^,^^67^ These results further provide evidence toward a process model across individual notes, which has been similarly investigated in the context of tonality.^68^^,^^69^

In Exp. 2, we sought to understand the influence of certain geometric properties in modulating the effects observed in Exp. 1. We conducted a large-scale behavioral study to test sensitivity for note deviations across all available 66 five-note sets (in standard tuning; Figure 4). We find remarkable variability among the sets tested: while certain sets exhibited no significant influence on sensitivity, others elicited a response bias toward note deviations that is twice in magnitude to a baseline choice (Figure 5A). Interestingly, the 5-note set eliciting the weakest bias toward note deviations was not significantly different from the chromatic set tested in Exp. 1. This indicates that this effect cannot be solely attributed to the number of notes in the set (Figure 5B). As such, working memory demands, which may be associated with set cardinality, do not appear to be a determining factor in explaining this distribution.^70^

Previous theoretical accounts propose that sets that exhibit a more even distribution within the octave minimize perceptual ambiguity in recognizing individual notes.^46^ This property has also been proposed to enhance perceptual sensitivity toward rhythmic deviations.^71^^,^^72^ Indeed, we found that evenness significantly correlates with the overall response bias toward note deviations. In the visual domain, recent studies have implicated symmetry as a critical feature of shapes that aids in perceptual processing.^10^^,^^20^^,^^21^ As such, our findings support the notion that geometric regularity is an organizing principle of mental representation across sensory domains.^5^^,^^10^

Another central feature of a musical set is the structure formed by its intervallic relationships (^46^^,^^73^; Figure 1). We find that a count of the set’s intervallic content is strongly predictive of the overall response patterns in Exp. 2. Specifically, the number of IC5s (perfect 5^th^s and their complementary interval, the perfect 4^th^) captures the greatest amount of variance relative to other interval classes, suggesting that IC5s facilitate sensitivity toward a unified note collection. This finding aligns with corpus analyses indicating that sets that maximize IC5s are more prevalent cross-culturally.^12^^,^^45^^,^^74^ Furthermore, our findings are corroborated by recent experimental work showing that melodies containing simple frequency ratios, including perfect fifths, enhance melodic processing abilities.^75^ Cross-cultural studies with indigenous Amazonian populations have demonstrated that the perceptual fusion of musical notes following simple frequency ratios (particularly octaves and perfect fifths) appears to be universal rather than culturally learned,^59^ possibly pointing to a mechanism for this advantage. IC5s further maximize the distribution of notes within an octave and reduce overall dissonance.^18^^,^^76^

It is important to mention that the mathematical nature of the set guarantees an inverse correlation between the number of IC5s and IC6s (tritones), possibly explaining the significant negative relationship we find between IC6 and sensitivity to note deviations. The negative relationship with respect to IC1 (minor 2^nd^ and major 7^th^) may be due to the overall set containing a less evenly distributed representation. As is likely evident at this point, sets are composed of many related features that interact with one another in a complex manner. As such, each result is likely not contributing to the overall effect in isolation, but rather through an interplay with interrelated factors.

The third experiment was aimed at understanding whether these effects were present in the absence of a hierarchical organization.^31^^,^^34^^,^^67^^,^^77^ To test this hypothesis, we investigated the influence of perfectly even sets, which preclude tonal organization.^45^^,^^46^^,^^47^ Our results reveal that 4-note and 6-note even sets elicit a robust sensitivity toward note deviations. These findings suggest tonality is not a prerequisite for establishing a sense of note membership. As such, the geometry underlying musical scales may reflect a constituent mental representation, separate from higher-order organizational principles in music. This experiment additionally leads to several other important interpretations: First, the findings underscore the importance of evenness as a fundamental geometric property determining the efficacy of a set for establishing note membership. Second, since these sets contain no instances of IC5, this analysis invites the interpretation that these interval classes are not a necessary condition at the membership-granting stage, despite their established role in tonality.^77^

Finally, in the fourth experiment, we examined whether sets modulate perceptual abilities while minimizing the impact of familiarity with Western musical systems. Our findings revealed that the pentatonic set, instantiated within a tritave (3:1 frequency ratio), elicited a significantly greater bias toward note deviations compared to a 5-note control set. This underscores that a scale’s geometry can enhance perceptual sensitivity in the absence of familiarity. Furthermore, this experiment demonstrates that the relational geometry itself facilitates processing outside of an octave-based system. This relates to a broader literature examining participants' ability to engage with musical systems that are not constrained to an octave.^64^^,^^78^

**Figure 6:**
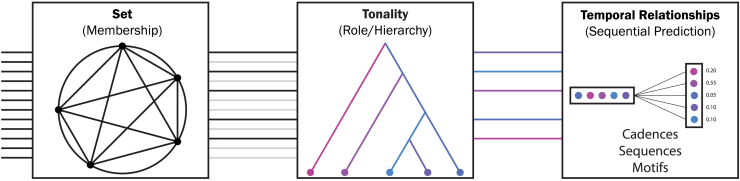
Situating the scale geometry in a process model of melodic processing In this process model, the first box represents how the 12 available pitches get filtered to include only those that are part of the scale. The remaining notes remain as secondary auxiliary notes (chromatic inflection notes) while the set’s notes represent the primary members underlying the subsequent steps in the model. The second box applies syntactic/tonal roles to the selected notes. In this step, a key and tonic are assigned along with other hierarchical roles to the rest of the set’s members. The 3rd box represents the specific temporal dynamics that are characteristic of a specific musical idiom, style, or genre. For example, it is this processing component that sets apart a blues melody from a traditional Chinese melody; while both idioms use the pentatonic set of notes with similar roles assigned, the way those roles unfold over time within the Chinese idiom is different than that of the blues. Having heard a series of notes in a particular sequence (horizontal bar), the next note is predicted with some probability (vertical bar). Once members are selected, roles are assigned, and temporal behaviors unfold, we end up with the perceptual experience of a musical melody that holds musical significance/meaning.

In conclusion, we demonstrate that scales establish a sense of membership, serving to facilitate melodic processing. Accordingly, we argue that these schematic structures represent an understudied construct in music perception that may constitute the basis for more complex musical representations and operations (Figure 6). We propose a processing model in which scales serve to facilitate a sense of note members, after which individual notes are attributed distinct musical roles (through tonality,^77^). Subsequently, these roles are processed with respect to their immediate temporal relationships and high-order temporal statistics, a phenomenon studied extensively within the predictive coding framework.^38^^,^^39^^,^^79^ As such, the concept of a scale, and the membership afforded by it, links concepts at distinct levels of explanation to extend a model of melodic processing. In summary, the model we argue for posits that scales determine membership, tonality determines roles, and temporal statistics provide the instantiation of those roles over time (Figure 6).

The findings of this work contribute to a broader research program seeking to understand complex mental structures across symbolic domains. This overarching goal has been a topic of considerable importance in mathematics,^20^^,^^21^ language,^6^^,^^7^ and music.^67^^,^^80^ Studying the structure of mental representations through the vantage point of geometry has contributed key recent insights into their role in these domains.^10^ As such, the current work contributes to this research program by providing a geometric account of a specific instance of mental representation in melodic processing. This renders this form of structural knowledge particularly well-suited for cross-domain comparison. Such work could reveal domain-general principles and constraints that govern the structure and informational content of internal representations.

### Limitations of the study

This study does not control for participant’s musical backgrounds in terms of familiarity and fluency in non-Western musical genres. For this reason, additional work will be needed to understand how experience with non-Western music will impact the findings reported in the present study.

Notably, theoretical accounts generally consider sets as composed of pitch classes that remain invariant across octaves, based on the perceptual principle of octave equivalence.^43^^,^^66^ In this study, we conceptualize sets within the confines of a single span (octave or tritave in Exp. 4), deliberately avoiding assumptions about their invariance across spans. In several decades of empirical research, octave equivalence as a perceptual construct has been largely the consensus. However, recent research has revealed significant cultural and individual differences in the perception of octave equivalence.^49^^,^^78^ Future studies will be needed to understand the impact of these schematic structures across octave spans at the level of individual melodies.

## Resource availability

### Lead contact

Further information and requests for resources should be directed to and will be fulfilled by the lead contact, Omri Raccah (omri.raccah@yale.edu).

### Materials availability

This study did not generate new unique reagents.

### Data and code availability

• All data in this paper are available at OSF (https://osf.io/phj8m/).
• All original codes have been deposited at OSF (https://osf.io/phj8m/) and are publicly available.
• Any additional information required to reanalyze the data reported in this paper is available from the lead contact upon request.

## Acknowledgments

This work was supported by a National Science Foundation Graduate Research Fellowship (NSF-GRFP) to O.R. (DGE 1839302).

We thank Poeppel Lab members, Aniruddh Patel, Morwaread Farbood, Neils Verosky, Aaron Kucyi, Erica Busch, and Daniel Brennan, for their comments and support.

## Author contributions

O.R. and M.S. conceived of the study. O.R. and M.S. designed and implemented the experiments. O.R., M.S., and C.P. analyzed the data. O.R. and M.S. prepared the article. All authors edited the article. F.L. and D.P. provided supervision for all aspects of the study.

## Declaration of interests

The authors declare no competing interests.

## STAR★Methods

### Key resources table

**Table:** 

REAGENT or RESOURCE	SOURCE	IDENTIFIER
**Deposited data**		

Stimulus and data	Open Science Framework (OSF)	https://osf.io/phj8m/
Experiment and analysis code	Open Science Framework (OSF)	https://osf.io/phj8m/

**Software and algorithms**		

jsPsych 6.1.0	De Leeuw et al.^81^	https://www.jspsych.org
Python 3.9	Python Software Foundation	https://python.org
EDO.js 1.2.16	Seltenreich^48^	https://michaelsel.github.io/edoJS/
Scikit-learn 0.23.2	Pedregosa et al.^82^	https://scikit-learn.org
SciPy 1.16.1	Virtanen et al.^83^	https://scipy.org

### Experimental model and study participant details

#### Participants for experiment 1

Three cohorts of participants were recruited from New York University to take part in the online experiment (N = 102). Participants were excluded if they reported not understanding the experiment in the post-task questionnaire were excluded (N = 4; three or less on a five-point Likert scale). The remaining participants (N = 98) were sequentially assigned to one of three cohorts, each exposed to one of three melody lengths (8-note, 12-note, and 16-note). The demographics for each cohort are as follows: 8-note (N = 28; 22 females; mean age 19.14 years, age range 18–21), 12-note (N = 29; 24 females; mean age 19.89 years, age range 18–22), and 16-note (N = 41; 35 females; mean age 19.07 years, age range 17–25). To ensure reliable measurement for each task condition in this online experiment, we excluded conditions that received fewer than 15 responses. This threshold allowed us to account for trials where participants reported no difference between test melodies. The results of this experiment are consistent at this threshold (see Figure S5). One response from the chromatic set was excluded based on this criterion. The experiment was self-paced and lasted approximately one hour. All participants provided informed consent prior to experimental testing and were given course credit for completing the experiment. The study was approved by the local institutional review board (New York University’s Committee on Activities Involving Human Subjects).

#### Participants for experiment 2

Participants were recruited from New York University to take part in the online experiment (N = 742). Participants were excluded if they reported not understanding the experiment in the post-task questionnaire were excluded (N = 44; three or less on a five-point Likert scale). Task conditions that received fewer than 15 responses were excluded to ensure a reliable measurement for each test condition. This resulted in the exclusion of 130 participants who did not meet this criterion for the six sets administered. The remaining participants were used in subsequent analyses (N = 568; 387 females; mean age 19.56 years, range 17-54). The upper age limit was due to a participant aged 54; not including this individual, the age range would be 17-27. Some of the participants took the task multiple times, each time interacting with an unseen task set (9 participants took the task 3 times, 44 participants took the task 2 times, and the remaining took the task once). The online experiment was self-paced and was designed to last approximately one hour. All participants provided informed consent prior to experimental testing. Participants were given course credit for completing the experiment. The study was approved by the local institutional review board (New York University’s Committee on Activities Involving Human Subjects).

##### Participants for experiment 2b

Participants were recruited from New York University to take part in the online experiment (N = 157). Participants who reported they didn’t understand the experiment in a post-task questionnaire were excluded (N = 10; three or less on a five-point Likert scale). The remaining participants were included in subsequent analyses (N = 147; 95 females; mean age 19.48 years, age range 18–27). The online experiment was self-paced and was designed to last approximately one hour. All participants provided informed consent prior to experimental testing. Participants were given course credit for completing the experiment. The study was approved by the local institutional review board (New York University’s Committee on Activities Involving Human Subjects).

#### Participants for experiment 3

Participants (N = 128) were recruited from New York University to take part in the online experiment. Participants who reported they didn’t understand the experiment in a post-task questionnaire were excluded (reporting three or less on a five-point Likert scale; N = 13). Task conditions that received fewer than 15 were excluded from subsequent analyses to ensure a reliable measurement for each condition. This resulted in the exclusion of 2 participant. The remaining participants were split into two cohorts that each engaged with the task in 12-note melodies (N = 49; 32 females; mean age 20 years, range 18-24) or 16-note melodies (N = 64; 49 females; mean age 19.51 years, range 18-23). The online experiment was self-paced and was designed to last approximately one hour. All participants provided informed consent prior to experimental testing. Participants were given course credit for completing the experiment. The study was approved by the local institutional review board (New York University’s Committee on Activities Involving Human Subjects).

#### Participants for experiment 4

Participants were recruited from New York University to take part in the online experiment (N = 40). We excluded participants who reported they did not understand the experiment in a post-task questionnaire (reporting three or less on a five-point Likert scale; N = 5). Task conditions that received fewer than 15 responses were excluded from subsequent analyses. This criterion did not result in the exclusion of any participants. The remaining participants were used in subsequent analyses (N = 35; 22 females; mean age 19.68 years, range 18-23). The experiment was self-paced and was designed to last approximately one hour. All participants provided informed consent prior to experimental testing and were given course credit for completing the experiment. The study was approved by the local institutional review board (New York University’s Committee on Activities Involving Human Subjects).

### Method details

#### Experiment 1

##### Stimulus generation

The audio files were created using the default settings of the Pianoteq6 Standard software (modartt.com/pianoteq_overview; ver. 6.1.1). The duration of each note was 250ms with an 83ms gap between notes, resulting in a pace of 3 notes per second. The probe melodies were generated using a pseudo-random procedure over the members of a particular musical set (using the EDO.js package.^48^; For example, a melody generated to conform to the pentatonic set was constrained to only include notes belonging to that set (02479 in normal order) or one of its modes (0257T, 0358T, 02579, and 0357T). The random-walk procedure avoided note repetitions across consecutive notes confined the entire melody to the range of a single octave and avoided pitch leaps (i.e., larger than 6 semitones between consecutive notes). The parameters used in the EDO.js package, melody_generation function, were as follows: length = [cohort dependent: 8, 12 or 16], range = [0,12], repetition_minimal_gap = 1, mode = [set dependent: as described above], avoid_leaps_over = 6 (semitones). Following the generation of the probe melody, two test melodies were generated. One test melody was identical to the probe with the exception that its two middle notes were swapped in their order (notes 4/5 were exchanged in 8-note melodies, 6/7 in 12-note melodies, and 8/9 in 16-note melodies). The other test melody was identical to the probe melody with the exception that one of the two middle notes was shifted either up or down by a semitone. The shift direction was counterbalanced within each block. The shifted note was always a note that did not appear in the probe melody. In cases where such a shift was not possible, a new set of probe and test melodies was recursively generated until all conditions were satisfied. To engage with the relational structure formed by the pitch collections rather than the pitch frequencies themselves, a random transposition (counterbalanced across blocks) was then applied to all three melodies. The highest possible transposition resulted in melodies in the range of F#4 and F#5 and the lowest possible transposition resulted in melodies in the range of F#3 and F#4. Audio files were mono-encoded at 32-bit resolution and 44.1-kHz sampling rate and were compressed into the mp3 file format.

##### Task procedures

The experiment was conducted online using the jsPsych JavaScript library (^81^; version 6.1.0). Participants were instructed to use headphones or earphones for the duration of the experiment. The volume was controlled by the participants who were instructed to pick a comfortable audio level. The instructions were displayed on the participants’ screens and participants’ responses were collected on their native computers or external keyboards. Participants were administered a total of 120 trials, each consisting of a probe melody followed by two test melodies. Half of all trials were generated from the pentatonic set, while the other half was generated from the chromatic set. The order of the trials was randomized across participants. Trials were distributed over 6 blocks consisting of 20 trials split evenly across conditions (i.e., pentatonic set and chromatic set). In each trial, the words “Test Melody” appeared on the screen while the probe melody was presented. Following the probe melody, the screen displayed “Option 1” and “Option 2” when each of the test melodies was presented. The order in which melodies included the contour or note violation was counterbalanced in each block. The three melodies were played in succession separated by a 1000ms inter-trial interval. After hearing all three melodies, participants were instructed to indicate “Which of the two comparison melodies (Option 1 or Option 2) sounded more DIFFERENT than the test melody?” Participants were instructed to press [A] on their keyboard if “Option 1 was more different”, [L] if “Option 2 was more different”. Alternatively, they could press [G] to indicate they “were unable to pay attention or could not hear a difference between the comparison melodies”.

##### Post-task questionnaire

After participants completed the experiment, a questionnaire was administered to assess participants’ understanding of the task and musical expertise. The first two questions serve to assess subjects’ understanding and subjective difficulty of the task: “I understood the instructions of the task” and “I found the task difficult”. Participants were instructed to respond to statements on a Likert scale, which included five balanced responses (strongly disagree, disagree, neither agree nor disagree, or strongly agree). Next, we asked participants to provide autobiographical information about their musical experience. Participants were asked to indicate how many years of formal musical training they had received and to indicate the age range during which this training occurred. Additionally, participants were asked to specify whether they play a musical instrument and, if so, to indicate the instrument. Finally, participants were provided with an open-ended format to respond to the following questions: “What general strategy (if any) did you employ to assess similarity across the melodies?” and “Did you notice any specific changes in the music? If yes, please describe those changes.”

#### Experiment 2

##### Stimulus generation

This experiment used the same stimuli generation procedure as Experiment 1. However, melodies were constrained to all possible 5-note sets in standard tuning as opposed to exclusively the pentatonic and chromatic sets in Exp. 1. To this end, a list of all possible 5-note sets was generated using the EDO.js package (*get.scales* function.^48^; In total, there are 792 combinations of 5 notes in standard tuning (“12-choose-5”): $\left(\begin{array}{l} 12\\ 5 \end{array}\right)=792$. However, since our experiment is aimed at understanding the relational aspect of sets, we remain agnostic to transpositions. Therefore, all 12 transpositions of a set are considered as a single instance of a musical set structure, resulting in a total of 66 unique sets $\left(\frac{792}{12}\right)=66$. The probe and test melodies were generated using the same pseudo-random procedure as described for Exp. 1.

##### Task procedures

Participants were administered 6 of the 66 total sets, consisting of 20 trials each. We ensured that each set was administered to approximately an equal number of participants. The task followed the same procedure as Exp. 1 (see *Exp. 1 Task Procedure* section), including the same number of total trials distributed evenly across six blocks. Upon completing the task, an additional block consisting of 20 trials was run to estimate participants’ performance on the pentatonic set, when only a note deviation condition was included with the other condition being identical to the probe, serving as a ground truth condition. This data was not analyzed in the current work.

##### Experiment 2b: Subjective ratings across sets

Participants were instructed to respond to statements on a Likert scale, which included five balanced responses (strongly disagree, disagree, neither agree nor disagree, or strongly agree). Each participant was administered 6 (out of the 66 possible sets), with 20 trials per set. Participants indicated their answers for three statements: (1) “Some notes felt more important than others”; (2) “The audio clip was melodic”; (3) “The melody as a whole or part of it felt familiar.” The first statement was intended to evaluate whether participants felt a sense of hierarchy between notes in the melody, the second was assessing roughness, while the final question assessed how familiar melodies sounded to participants. The questions were shuffled across each trial in the task.

#### Experiment 3

This experiment used the same stimuli generation procedure as Experiment 1. However, melodies were constrained to the fully diminished 7^th^ (0 3 6 9) set and the whole-tone set (0 2 4 6 8 10), see Figure 5E. The task procedures followed the same protocols as Experiment 1, with trials distributed over 6 blocks consisting of 20 trials split evenly across conditions (60 trials per set condition). Furthermore, the randomization procedures were identical to Exp. 1. In this experiment, we only tested participants’ performance on 12-note melodies.

#### Experiment 4

To test the influence of scale geometry in an unfamiliar tuning system, we constructed melodies from sets instantiated along a tritave (3:1 frequency ratio). We divided the tritave into 12 equal parts in order to construct equivalent sets to those used in prior experiments. Specifically, we instantiated the pentatonic set (0 2 4 7 9) within this system. In this way, the relational structure underlying this scale remained identical to prior experiments while utilizing unfamiliar interval and note identities. As a control, we instantiated the lowest preforming set from Exp. 2 within this tuning system (0, 1, 2, 4, 8). The melodies were generated using the same pseudo-random walk procedures as in the previous experiments. The task procedures followed the same protocols as Exp. 1, with trials distributed over 6 blocks consisting of 20 trials split evenly across conditions (60 trials per set condition). Furthermore, the randomization procedures were identical to Exp. 1. In this experiment, we only tested participants’ performance on 12-note melodies.

### Quantification and statistical analysis

#### Quantifying set evenness

We measure how evenly distributed pitches are within the span of an octave (i.e., set evenness). This was computed by calculating the pairwise differences between the notes of a given set and those of a perfectly distributed 5-note set. After this, the standard deviation was computed over the differences as a measure of set evenness. As such, sets manifesting larger standard deviation from a perfectly distributed set were deemed “less even”, and those more closely resembling the perfectly distributed set were deemed “more even”.^84^

#### Estimating the effect of set intervals on perceptual sensitivity

The relationship between a set’s interval vector (IC1, IC2, etc.) and sensitivity to note deviations was assessed using a multiple ridge regression. The model was implemented in the Scikit Learn package (scikit-learn.org; version 0.23.2^82^; in Python (3.7.9), and was initialized with a penalty coefficient of 1.0. The count of each interval class served as an independent variable. The average bias toward note deviations across all 66 sets was the dependent variable. The performance of the full model was assessed using an R coefficient of the model predictions on the true values against a null distribution using a nonparametric permutation test. Beta coefficients for each interval class were similarly compared to a null distribution using a permutation test. We implemented 10,000 permutations to ensure a reliable estimate of the null distribution. Confidence intervals (95%) for regression coefficients were derived from the permutation distributions. Statistical significance was evaluated at *p* < 0.05.

#### Statistical analysis

All statistical analyses were conducted using Python (version 3.7.9) packages, including SciPy^83^ (version 1.16.1), Statsmodels^85^ (version 0.14.1), Pingouin^86^ (version 0.5.3), and Scikit-learn^82^ (version 0.23.2). Statistical significance was evaluated at p < 0.05, and all tests were two-tailed unless specified otherwise. Statistical significance was evaluated at *p* < 0.05, and all tests were two-tailed unless otherwise specified.

In Exp. 1, we used a two-way mixed analysis of variance (mANOVA) with condition type (pentatonic vs. chromatic) as the within-subjects factor and melody length (8, 12, or 16 notes) as the between-subjects factor. Across all experiments, sensitivity to note deviations was quantified as the signed difference between response rates for note violations and contour violations. Within-condition effects were assessed using one-sample t-tests against zero, between-condition comparisons used paired t-tests, and cross-experiment comparisons employed independent samples t-tests. In Exp. 2, nonparametric permutation tests were used to evaluate significance for the overall model performance and individual predictors (10,000 permutations, *p* < 0.05; details for the interval vector analysis are provided in the proceeding section).
