# Supplementary material for: Geometric properties of musical scales constitute a representational primitive in melodic processing
Source: iScience. 2025 Oct 9;28(11):113701. doi: 10.1016/j.isci.2025.113701 (PMC12682281; doi:10.1016/j.isci.2025.113701)
Supplement: Document S1. Figures S1–S5 and Table S1 [file mmc1.pdf]

iScience, Volume *28*

Supplemental information

Geometric properties of musical scales constitute a representational primitive in melodic processing

Omri Raccah, Michael Seltenreich, Claire Pelofi, Fred Lerdahl, and David Poeppel

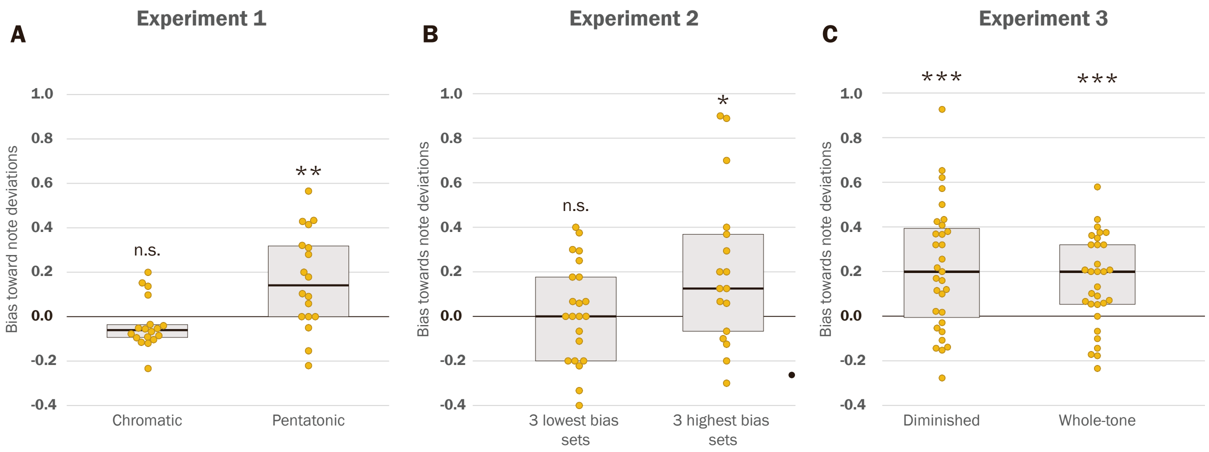

Figure S1. Performance of non-musicians across experiments. Data from participants with no formal years of musical training or experience playing a musical instrument were analyzed. (A) In Exp. 1, non-musicians, aggregated across 12-note and 16-note cohorts, showed a significant preference for note deviations in the pentatonic set and no significant preference in the chromatic set. (B) In Exp. 2, non-musicians exhibited a significant bias towards note deviations in the highest bias sets and no significant effects were observed for the lowest bias sets. (C) In Exp. 3, non-musicians, aggregated across 12-note and 16-note cohorts, demonstrated a significant bias towards note deviations in both the diminished and whole-tone sets. One-sample t-test. * $p < 0.05$. ** $p < 0.01$. *** $p < 0.001$

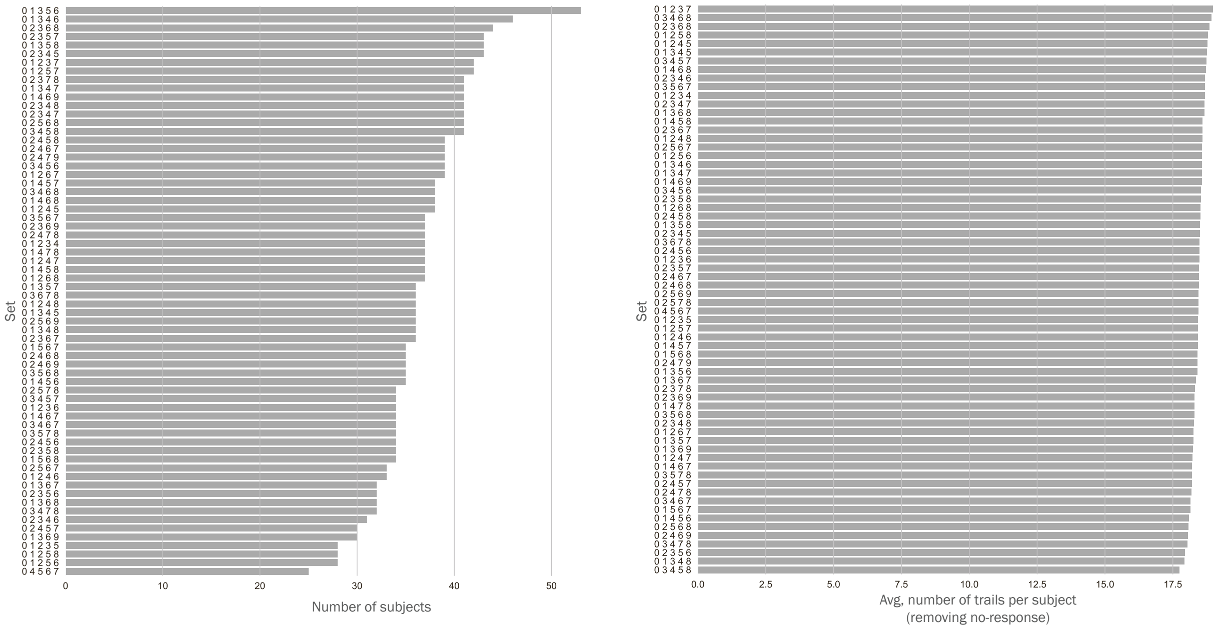

Figure S2. Number of participants and trials analyzed in Experiment 2 by musical set. (Right) The number of participants that were tested for each set structures. (Left) the average number of trials per participant after excluding trials in which participants indicated no difference between test melodies. As shown, these values were approximately the same across all sets tested in the experiment.

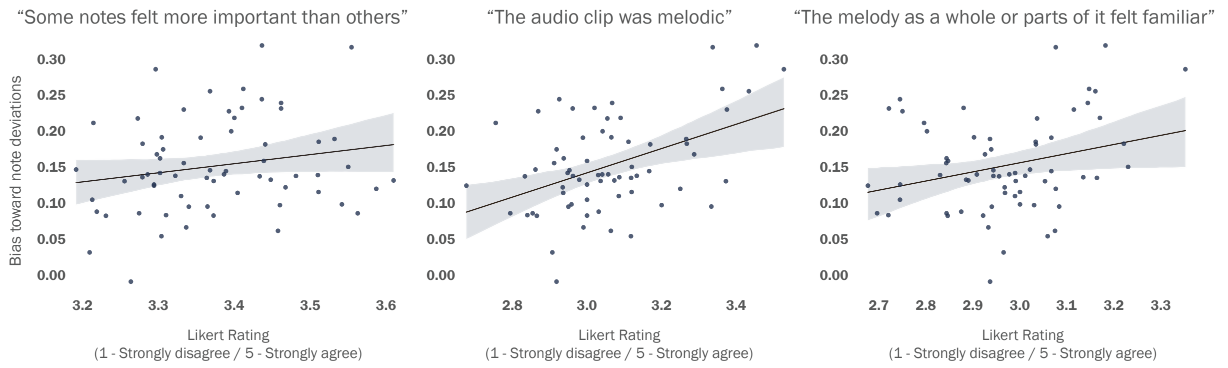

Figure S3. Subjective ratings and relationship with behavioral effects in Experiment 2. Likert scale ratings were collected for each of the 66 sets in Experiment 2 from a separate cohort (N = 147, post-exclusion; see Materials and Methods section). For each set, we calculated the average of the median ratings for the three questions. Participants' subjective ratings of how melodic the stimuli sounded were significantly correlated with the bias for note deviations ($R = 0.45$, $p < 0.001$; Pearson correlation). We found no significant correlation between familiarity and the bias toward note deviations ($p > 0.05$). Similarly, no significant was found between the importance of some notes over others and the bias toward note deviations ($p > 0.05$).

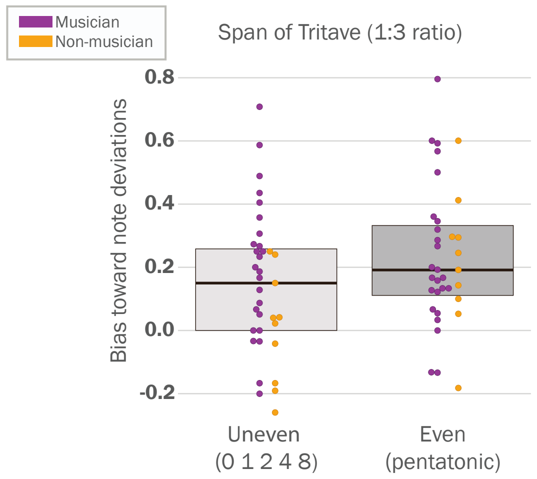

Figure S4. Experiment 4 results: response bias in sets instantiated in a tritave. We compared response bias for a pentatonic set embedded in a tritave (pentatonic-UT) and a control 5-note set embedded in a tritave (control-UT). the pentatonic-UT elicited a significantly greater bias toward note deviations compared to the control set ($t(34) = 2.67$, $p = 0.012$, Cohen’s $d = 0.38$; paired t-test). This effect was more pronounced when examining only non-musicians. Paired t-test ($t(10) = 3.41$, $p = 0.008$, Cohen’s $d = 1.07$; paired t-test).

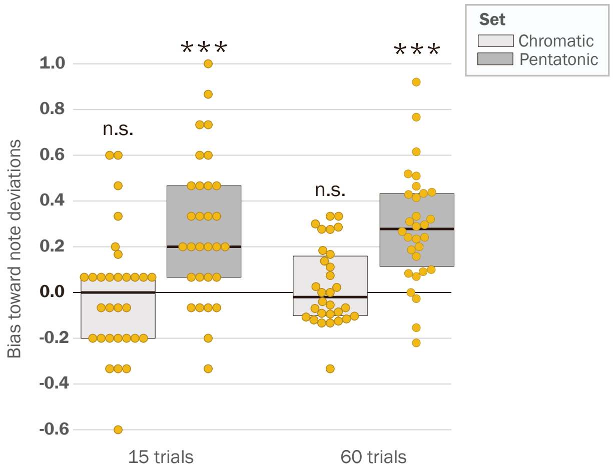

Figure S5. Establishing a threshold for excluding task conditions with few responses. We excluded task conditions with fewer than 15 responses to account for trials in which participants reported no difference between tasks. We analyzed the data specifically from 12-note melodies as this length is used for all subsequent experiments. At this threshold, we were able to replicate the findings from Exp. 1 (pentatonic 15-responses: $t(28) = 5.53$, $p < 0.00$, Cohen's $d = 0.98$; pentatonic 60 or all-responses: $t(28) = 6.04$, $p < 0.001$, Cohen's $d = 1.12$). Consistent across both response quantities, we find no significant bias toward note deviations for the chromatic set ($p > 0.05$). One-sample t-test. *** $p < 0.001$.

Table S1. Evenness, intervallic counts, and scores across the sets tested in Experiment 2.

Set	Bias towards note deviation	IC1	IC2	IC3	IC4	IC5	IC6	Evenness
0 1 2 4 8	-0.0080	2	2	1	3	1	1	0.9057
0 1 2 3 6	0.0327	3	2	2	1	1	1	0.8731
0 1 2 3 7	0.0552	3	2	1	1	2	1	0.8821
0 3 4 6 8	0.0627	1	2	2	3	1	1	0.9333
0 4 5 6 7	0.0675	3	2	1	1	2	1	0.8821
0 1 2 6 7	0.0836	3	1	0	1	3	2	0.9150
0 3 5 6 7	0.0839	2	2	2	1	2	1	0.9028
0 1 2 4 6	0.0845	2	3	1	2	1	1	0.8894
0 2 5 6 8	0.0870	1	2	2	2	1	2	0.9423
0 2 4 5 6	0.0871	2	3	1	2	1	1	0.8894
0 1 2 5 7	0.0893	2	2	1	1	3	1	0.9150
0 1 2 3 5	0.0962	3	3	2	1	1	0	0.8566
0 2 5 6 7	0.0965	2	2	1	1	3	1	0.9150
0 1 4 5 8	0.0984	2	0	2	4	2	0	0.9376
0 1 2 6 8	0.0995	2	2	0	2	2	2	0.9255
0 2 4 5 8	0.1059	1	2	2	3	1	1	0.9333
0 2 3 4 5	0.1110	3	3	2	1	1	0	0.8566
0 1 3 5 7	0.1154	1	3	1	2	2	1	0.9255
0 2 3 5 6	0.1167	2	2	3	1	1	1	0.8920
0 1 3 5 6	0.1211	2	2	2	1	2	1	0.9028
0 2 3 6 9	0.1232	1	1	4	1	1	2	0.9473
0 1 5 6 7	0.1255	3	1	0	1	3	2	0.9150
0 1 4 6 9	0.1270	1	1	3	2	2	1	0.9592
0 1 3 4 6	0.1317	2	2	3	1	1	1	0.8920
0 1 4 6 8	0.1320	1	2	1	3	2	1	0.9529
0 1 4 5 7	0.1327	2	1	2	2	2	1	0.9218
0 1 3 4 5	0.1338	3	2	2	2	1	0	0.8688
0 1 2 4 7	0.1363	2	2	2	1	2	1	0.9028
0 2 3 4 7	0.1372	2	2	2	2	2	0	0.8973
0 2 4 6 8	0.1385	0	4	0	4	0	2	0.9529
0 1 4 7 8	0.1390	2	0	2	3	2	1	0.9473
0 1 3 6 9	0.1392	1	1	4	1	1	2	0.9473
0 1 2 5 8	0.1402	2	1	2	2	2	1	0.9218
0 2 3 4 6	0.1411	2	3	2	2	0	1	0.8798
0 2 3 6 8	0.1412	1	2	2	2	1	2	0.9423
0 1 2 4 5	0.1431	3	2	2	2	1	0	0.8688
0 2 4 6 7	0.1457	1	3	1	2	2	1	0.9255
0 1 4 5 6	0.1469	3	1	1	2	2	1	0.8973

0 2 3 4 8	0.1479	2	2	1	3	1	1	0.9057
0 3 4 5 8	0.1516	2	1	2	3	2	0	0.9150
0 3 4 5 6	0.1569	3	2	2	1	1	1	0.8731
0 2 4 7 8	0.1599	1	2	1	3	2	1	0.9529
0 2 5 6 9	0.1635	1	1	3	2	2	1	0.9592
0 3 6 7 8	0.1691	2	1	2	2	2	1	0.9218
0 3 4 6 7	0.1760	2	1	3	2	1	1	0.9087
0 1 3 6 8	0.1829	1	2	2	1	3	1	0.9473
0 2 4 6 9	0.1838	0	3	2	2	2	1	0.9667
0 3 4 7 8	0.1865	2	0	2	4	2	0	0.9376
0 1 2 3 4	0.1904	4	3	2	1	0	0	0.8350
0 2 3 5 8	0.1922	1	2	3	1	2	1	0.9293
0 1 3 6 7	0.1927	2	1	2	1	2	2	0.9293
0 2 5 7 8	0.2011	1	2	2	1	3	1	0.9473
0 1 3 4 7	0.2127	2	1	3	2	1	1	0.9087
0 3 4 5 7	0.2190	2	2	2	2	2	0	0.8973
0 1 5 6 8	0.2197	2	1	1	2	3	1	0.9376
0 1 4 6 7	0.2290	2	1	2	1	2	2	0.9293
0 3 5 7 8	0.2313	1	2	2	2	3	0	0.9376
0 1 2 5 6	0.2328	3	1	1	2	2	1	0.8973
0 2 3 6 7	0.2336	2	1	2	2	2	1	0.9218
0 2 3 7 8	0.2405	2	1	1	2	3	1	0.9376
0 3 5 6 8	0.2457	1	2	3	1	2	1	0.9293
0 2 3 5 7	0.2568	1	3	2	1	3	0	0.9150
0 1 3 4 8	0.2600	2	1	2	3	2	0	0.9150
0 2 4 5 7	0.2874	1	3	2	1	3	0	0.9150
0 1 3 5 8	0.3180	1	2	2	2	3	0	0.9376
0 2 4 7 9	0.3205	0	3	2	1	4	0	0.9764
